# Carbon Nanotube Film Electrodes with Acrylic Additives: Blocking Electrochemical Charge Transfer Reactions

**DOI:** 10.3390/nano10061078

**Published:** 2020-05-31

**Authors:** Alejandro Ansón-Casaos, Olga Sanahuja-Parejo, Javier Hernández-Ferrer, Ana M. Benito, Wolfgang K. Maser

**Affiliations:** Instituto de Carboquímica, ICB-CSIC, Miguel Luesma Castán 4, 50018 Zaragoza, Spain; osanahuja@icb.csic.es (O.S.-P.); jhernandez@icb.csic.es (J.H.-F.); abenito@icb.csic.es (A.M.B.); wmaser@icb.csic.es (W.K.M.)

**Keywords:** carbon nanotubes, acrylic additives, inks, conductive films, flexible electrodes, electrochemistry

## Abstract

Carbon nanotubes (CNTs) processed into conductive films by liquid phase deposition technologies reveal increasing interest as electrode components in electrochemical device platforms for sensing and energy storage applications. In this work we show that the addition of acrylic latex to water-based CNT inks not only favors the fabrication of stable and robust flexible electrodes on plastic substrates but, moreover, sensitively enables the control of their electrical and electrochemical transport properties. Importantly, within a given concentration range, the acrylic additive in the films, being used as working electrodes, effectively blocks undesired faradaic transfer reactions across the electrode–electrolyte interface while maintaining their capacitance response as probed in a three-electrode electrochemical device configuration. Our results suggest a valuable strategy to enhance the chemical stability of CNT film electrodes and to suppress non-specific parasitic electrochemical reactions of relevance to electroanalytical and energy storage applications.

## 1. Introduction

Carbon nanotubes (CNTs) show remarkable electrical conductivity and specific surface area. Processed into CNT films, they reveal great promise as electrode materials for various applications such as resistive gas sensors [1,2,3] or electrochemical sensors, achieving high sensitivity and selectivity for the detection of inorganic cations [4,5] and organic molecules [6,7,8,9]. Besides, conducting electrodes and interfaces made of CNTs or containing CNT additives have been developed for electrochemical energy harvesting and storage devices [10,11,12,13,14,15]. In particular, the great potential of CNTs for flexible device structures was quickly identified [16,17]. In all the mentioned electrochemical applications, it is necessary to prevent undesired charge transfer reactions, which lead to a decrease in the efficiency, low selectivity, increased corrosion processes, and decreased device lifetimes.

Active CNT components and electrode layers are commonly fabricated by liquid phase processing of CNT inks, such as drop-casting, printing or spray-coating, among others. In this regard, water-based dispersions are desirable as an eco-friendly strategy, instead of organic solvents. In the literature, water-based conducting inks have been prepared by simply mixing graphene and chemically functionalized multi-walled carbon nanotubes (MWCNTs) [18]. However, as carbon nanomaterials are in principle hydrophobic, inks for printed electronics are most often stabilized by a surfactant, such as sodium dodecylbenzenesulfonate (SDBS) or polyvinylpyrrolidone [19,20,21]. Once printed or painted, typical difficulties for the utilization of CNT films are their low adhesion, in particular to flexible plastic substrates, and their sensibility to mechanical damage. Pure CNT films quite easily crack or break during deposition, transfer, immersion in liquids, and general handling. Methods for improving the mechanical resistance of CNT films include CNT functionalization, chemical post-treatments [22,23], and fabrication under controlled atmospheres [24]. Another interesting solution is the addition of an acrylic binder to water-based CNT dispersions. Moreover, these acrylic inks can be deposited on flexible plastic substrates, for instance by spray-coating [25] or rod-coating [26].

Therefore, the addition of certain quantities of acrylic resins has been shown to be compatible with the fabrication of conductive CNT films [25,26]. Later on, acrylic inks containing graphene and CNTs have been processed by gravure printing, achieving high electrical conductivity for printed electronics [27]. In addition, acrylic inks containing functionalized MWCNTs have been applied to the fabrication of electroluminescent devices, capacitive sensors, and wearable electronics by screen printing, showing high electrical conductivity, mechanical stability, and even resistance to folding [28]. In electrochemical applications, acrylic MWCNT inks have been deposited on glassy carbon electrodes, resulting in improved electrocatalytic activities [29,30]. Moreover, flexible electrodes made of the acrylic ink have been used as support for enzyme immobilization with remarkable biosensing properties [29].

However, the effects of acrylic additives on the electronic transport through the CNT/solution interface have not been analyzed in detail, to our knowledge. In this work, we study the joint control of electric conductivity, capacitance, and faradaic transfer properties of CNT electrodes prepared from acrylic inks. The concentration of the acrylic component must be optimized in a well-defined range to keep the electrical conductivity and the CNT external surface, while preventing parasitic redox reactions. The knowledge provided in this work brings significant advantages to the fabrication of electrodes for electrochemistry and components for electronic and energy devices.

## 2. Materials and Methods

### 2.1. Materials

MWCNTs (NC 7000 grade) were purchased from Nanocyl (Sambreville, Belgium). The NC 7000 powder material contained <10 wt. % of metal oxides, and MWCNTs had an average diameter and length of 9.5 nm and 1.5 μm, respectively, according to the provider. The complete characterization of the solid, including elemental analysis and Raman spectrum, as well as its dispersion properties, have been previously published [31,32].

The SDBS surfactant was purchased from Sigma-Aldrich (Madrid, Spain). The acrylic resin Hycar^®^ 26084 was provided by Lubrizol Advanced Materials (Sant Cugat del Vallés, Barcelona, Spain). Hycar^®^ 26084 is a selfcrosslinking modified carboxylated acrylic latex that bonds to a great variety of plastic substrates. It has the appearance of a milky white emulsion. The solid dry mass of Hycar^®^ was determined by evaporation in a plate, resulting in 437.8 mg·mL^−1^ or 43.8 wt. %.

### 2.2. Ink Preparation and Electrode Deposition

MWCNTs (8 mg·mL^−1^) were dispersed in a 5 mg·mL^−1^ SDBS aqueous solution by tip sonication. Definite volumes of the dispersion were diluted with deionized water and mixed with different volumes of the acrylic latex (Table 1). In this way, different sample dispersions, which are hereafter called MSH-*i* (*i* = 0–4, Table 1), were considered, depending on the MWCNT/acrylic resin ratio.

Rheological measurements were performed using a Kinexus Ultra rotational rheometer (Malvern, Worcestershire, United Kingdom). The experimental configuration was a cone and plate of 50 mm diameter and an angle of 2 degrees. The temperature was kept at 25 °C, and the experimental range was 0.01–100 s^−1^ under shear rate control.

The ink was spray-coated (~2 mL·cm^−2^) using a manual spray gun (Premium 475, Sagola, Vitoria, Spain) on small pieces of transparent polyethylene terephthalate (PET, 0.1 mm thick, Schwan Stabilo) heated to ~80 °C. Considering the total evaporation of liquids in the ink, the composition of the deposited films is included in Table 2.

### 2.3. Electrical and Electrochemical Characterization

Electrical resistance on the surface of CNT electrodes was measured at room temperature using a Keithley 4200-SCS system (Keithley, Cleveland, OH, USA). An in-line 4-point probe configuration with equidistant probe separations of 2.24 mm was utilized for measurements of surface resistivity and electrical conductivity. The specimen thickness was measured with an electronic digital caliper. The deposited mass was determined by direct weighing.

Electrochemical measurements were carried out in a three-electrode configuration using the Autolab PGSTAT 302N potentiostat (Metrohm AG, Herisau, Switzerland). The reference electrode was Ag/AgCl, 3M NaCl (*E*° = 0.210 V), and the counter electrode was a graphite rod. The CNT film was connected to the potentiostat probe through a copper wire and copper adhesive tape. Solutions of 0.1 M Na_2_SO_4_ (Sigma-Aldrich) and 0.1 M phosphate buffer (pH = 6.85, Sigma-Aldrich) were used as supporting electrolytes. Faradaic transfer properties were tested using three different redox couples: K_3_Fe(CN)_6_/K_4_Fe(CN)_6_ (Fisher Scientific, Madrid, Spain) in 0.1 M Na_2_SO_4_, and ascorbic acid and hydroquinone (Sigma-Aldrich) in 0.1 M phosphate buffer (pH = 6.85).

## 3. Results and Discussion

**Figure 1:**
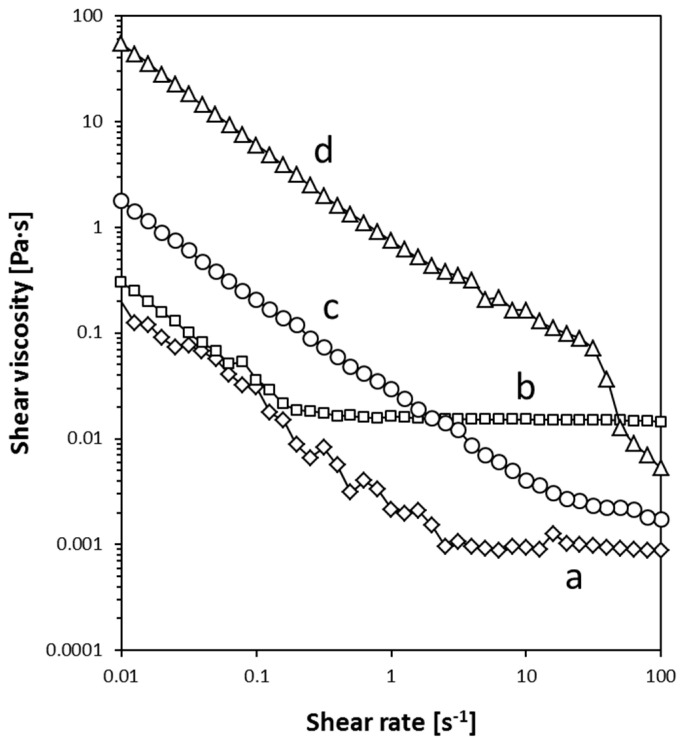
Rheological description of liquid products and dispersions: (**a**) SDBS solution; (**b**) Hycar^®^ 26084 acrylic resin; (**c**) MSH-2; and (**d**) MSH-0.

First of all, we briefly present an assessment of the rheological properties of CNT acrylic inks (Figure 1). The rheological information is relevant for ink processing, in this case by spray coating. Both the SDBS base medium and the Hycar^®^ acrylic resin showed Newtonian behaviors at shear rates above 2.5 and 0.2 s^−1^, respectively. The MSH-0, which bore 8 mg·mL^−1^ of MWCNTs in the pure SDBS solution, presented shear thinning in the whole measurement range up to 100 s^−1^. However, diluted inks such as MSH-2, which contained 4 mg·mL^−1^ of MWCNTs and increasing quantities of the Hycar^®^ acrylic resin, approached a Newtonian regime at shear rates above 25 s^−1^. The change from a nearly Newtonian liquid to the shear thinning behavior by increasing MWCNT concentrations has been previously described in the literature [33,34]. As a general comment on the rheological properties, it can be stated that the viscosity of the MWCNT ink is not substantially altered by the addition of certain quantities of the acrylic Hycar^®^. Therefore, inks with and without the acrylic component can be used under analogous processing conditions; in this work, all the inks were deposited by spray-coating to fabricate film electrodes.

**Figure 2:**
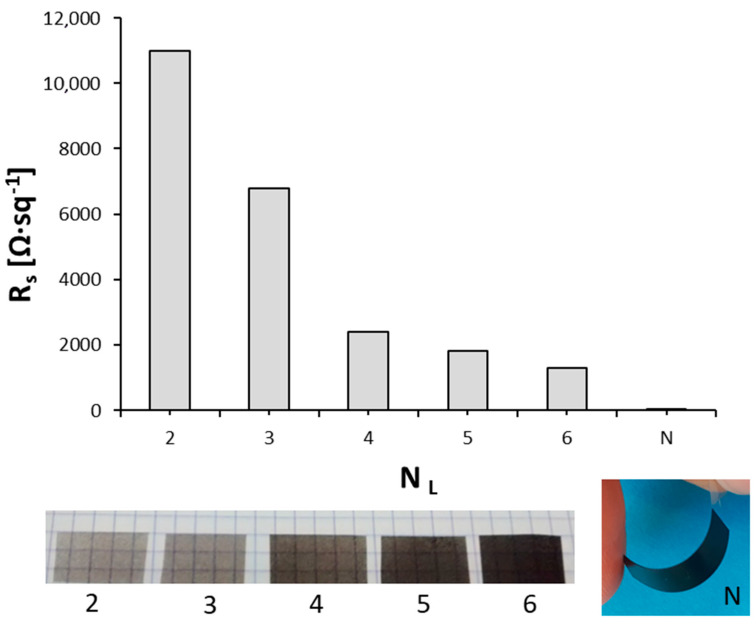
Sheet resistance (*R*_s_) of MSH-2 films with increasing number of layers (*N*_L_). The final electrode with a N layers had a thickness of 0.06 mm and R_s_ of 11 Ω·sq^−1^.

Next, the electrical transport properties of CNT films are described. Figure 2 shows the evolution of sheet resistance (*R*_s_) as the spray-coating process on PET films progressed. Images in Figure 2 indicate that CNT films remained semi-transparent until the deposition of 4–5 layers of the CNT ink, while they became opaque for thicker films. It can be seen that the films were well uniform and homogeneous. The *R*_s_ value was higher than 10^4^ Ω·sq^−1^ for 2-layer films, and decreased rapidly as the thickness increased. The final *R*_s_ value for an electrode with a thickness of 0.06 mm was in the range of 10 Ω·sq^−1^, which was highly competitive compared with other previously published carbon-based conducting inks [28]. Besides, the electrodes showed a high mechanical resistance, adhesion of the CNT film to the plastic substrate, and durability upon bending (Figure 2), in agreement with previous literature works [29].

The electrical conductivity (*σ*) of CNT composites, in particular acrylic composites, with increasing concentrations of the filler, followed a percolation behavior [35,36]. Acrylic polymers were typically resistive materials with very low electrical conductivities (*σ* < 10^−13^ S·cm^−1^). The *σ* value increased abruptly at a certain CNT loading, which is called the percolation threshold. Due to the extremely high aspect ratio of CNTs, the percolation threshold occurred at very low loadings, typically lower than 1 wt. % of CNTs. At the percolation threshold, the nanoparticles established a conducting network through direct or tunneling contacts [37,38], and thus the conductivity strongly increased by >10 orders of magnitude. However, the *σ* still increased progressively with further additions of the filler above the percolation threshold, as the number and width of transporting channels increased. Such increments are relevant for the preparation of conducting inks and electrodes, which typically require σ values of well above 1 S·cm^−1^.

**Figure 3:**
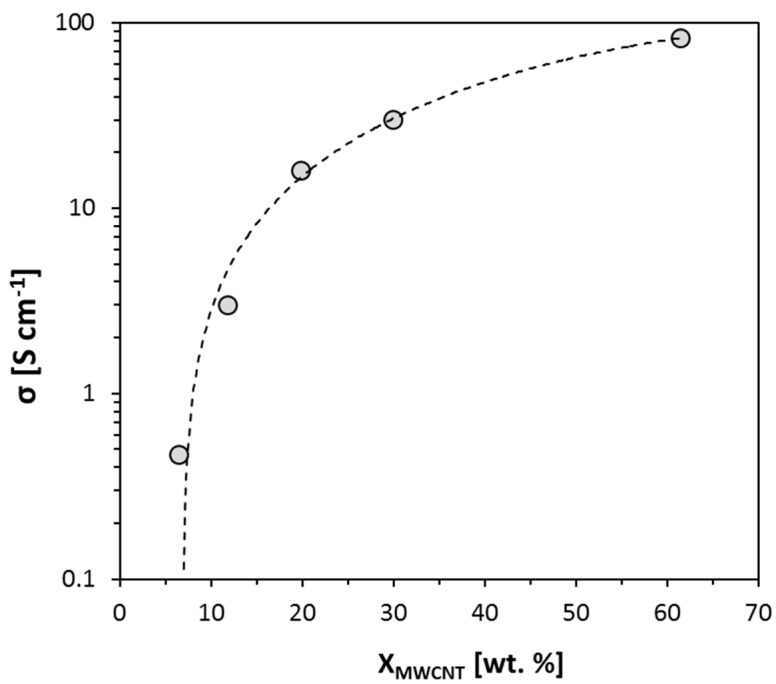
Electrical conductivity of films MSH-0 to -4 (Table 2) with various concentrations of MWCNTs on a dry basis.

The *σ* values for MWCNT electrodes with increasing concentrations of the Hycar^®^ 26084 acrylic component are listed in Table 3. The SDBS surfactant, in dry weight basis, had to be considered as an insulating component in the composite. The trend of σ with MWCNT concentrations (*X*_MWCNT_) above 5 wt. % is represented in Figure 3. Even though *X*_MWCNT_ was in all cases above the percolation threshold, the σ value still changed by 2 orders of magnitude in the range of 10 to 60 wt. %. For their use as electrodes, we focused on films with *X*_MWCNT_ > 20 wt. % (MSH-2, MSH-1 and MSH-0 specimens), and we analyzed their electrochemical response.

**Figure 4:**
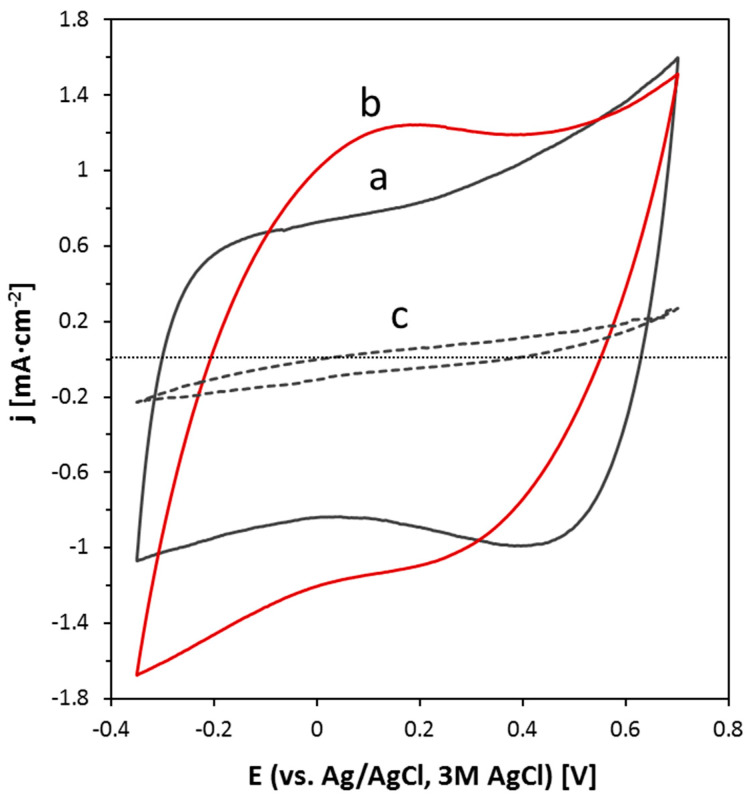
Cyclic voltammograms after 10 cycles in 0.1M Na_2_SO_4_ at 100 mV/s of three electrodes: (**a**) MSH-0; (**b**) MSH-1; and (**c**) MSH-2.

Figure 4 shows the analysis of MSH-0, MSH-1, and MSH-2 electrodes by cyclic voltammetry. The MSH-0 film presented a nearly rectangular voltammogram, with a substantial double layer capacitance (8 mF·cm^−2^). This is the typical behavior of CNTs in various electrode configurations, such as films, coatings, pellets, and buckypapers [39,40,41], in which the relatively high capacitance is associated with the high surface area of CNTs. Apparently, the presence of SDBS in the MSH-0 material did not prevent the capacitive response of CNTs. The addition of the Hycar^®^ acrylic resin up to ~50 wt. % of the mixture (MSH-1 specimen) produced certain changes in the general capacitive behavior of the composite electrode. Compared to MSH-0, the small decrease in the conductivity led to a somewhat leaning voltammogram in the measurement potential window (*E* = −0.3 and 0.7 V vs. Ag/AgCl, 3M AgCl). In addition, an increase in pseudo-capacitive currents, particularly in the cathodic branch of the voltammogram, was detected in the range of 0–0.4 V, leading to an overall capacitance of 8.6 mF·cm^−2^. It has to be noticed that the specific capacitance in mass basis decreased slightly in MSH-1, compared to MSH-0, due to the addition of Hycar^®^ (Table 3). The additional pseudo-capacitive signals were probably associated with oxygen functional groups on the acrylic resin, which was not thick enough to block the capacitive response of the MWCNTs in the film. 

While the general capacitive character of CNTs was kept in the MSH-1 specimen, the capacitance strongly decreased with further additions of the acrylic component (MSH-2 specimen, Figure 4, Table 3). When it was compared to MSH-1, the σ value only changed from 30 to 16 S·cm^−1^, while the capacitance very notably decreased (0.41 mF·cm^−2^). The strong drop in the capacitance, which was thus independent of the change in *σ*, could be assigned to the blocking of CNT electroactive surfaces by the acrylic component. Subsequent additions of the acrylic resin (MSH-3 and MSH-4 electrodes) led directly to a rather large decrease in *σ* values by more than one order of magnitude (Table 3); therefore, they were not used for the electrochemical assays, as already stated above.

**Figure 5:**
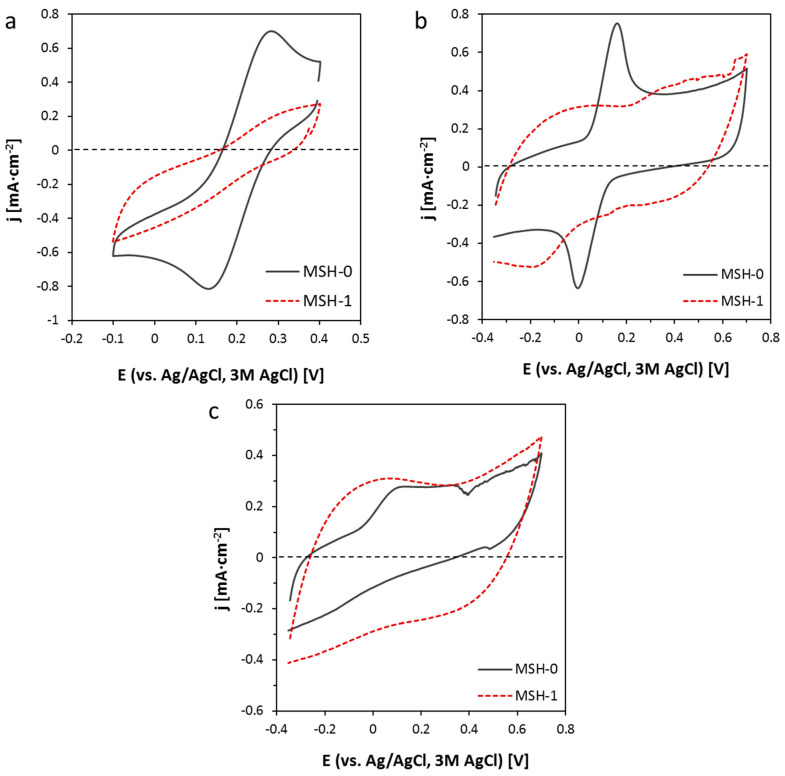
Cyclic voltammograms in the presence of three different redox couples: (**a**) K_3_Fe(CN)_6_/K_4_Fe(CN)_6_ in 0.1M Na_2_SO_4_ at 10 mV·s^−1^; (**b**) hydroquinone in 0.1M phosphate buffer, pH = 6.85, at 20 mV·s^−1^; (**c**) ascorbic acid in 0.1M phosphate buffer, pH = 6.85, at 20 mV·s^−1^.

In order to test the electrochemical faradaic charge transfer, further electrochemical characterization using redox couples was performed on those electrodes showing large electroactive surfaces, namely the MSH-0 and MSH-1. Figure 5 shows outcomes with the three tested redox probes. The charge transfer for the K_3_Fe(CN)_6_/K_4_Fe(CN)_6_ couple typically takes place through an outer sphere mechanism, while hydroquinone/benzoquinone and ascorbic acid require specific interactions with functional groups and aromatic rings on the CNT surface [42,43]. It could be observed that the presence of the acrylic component (MSH-1 electrode) drastically decreased electrode/electrolyte faradaic charge transfer currents in the three cases. 

In these electrodes, the rate constant *k*^0^ (Table 4), calculated combining Nicholson [44] and Mahé [45] methods, was strongly diminished by the presence of Hycar^®^. Nicholson’s equation was applied when the anodic–cathodic peak separation was <200 mV, while Mahé’s formalism covered the range of >200 mV. The huge peak separation for K_3_Fe(CN)_6_ reduction and hydroquinone oxidation (350 and 706 mV, respectively) on MSH-1 indicated the blocking of the interfacial electron transfer. This effect was achieved without blocking conductive and capacitive properties of the electrode, and it can be relevant to avoid CNT corrosion and other parasitic reactions in electrodes or interfaces for analytical, sensing, and energy devices. The *k*^0^ value decreased by one order of magnitude in the case of ferricyanide (1.84 × 10^−3^ vs. 1.62·× 10^−4^ cm·s^−1^), and even in a larger amount for hydroquinone (9.63 × 10^−4^ vs. 3.82 × 10^−6^ cm·s^−1^). The inhibition of the outer sphere electrochemical reaction indicated that the acrylic resin formed a barrier interface between CNTs and the electrolyte. Furthermore, as expected in this situation, adsorption on CNT active sites was soon inhibited, and reactions of ascorbic acid and hydroquinone even more markedly blocked. Apparently, functional groups in the acrylic component were not active towards faradaic charge transfer. Peak separation values for processes on MSH-0 were comparable to those obtained for CNT buckypaper electrodes [42].

The exact composition and physicochemical properties of acrylic latex additives varied depending on the supplier. Typical commercial products are made of methyl methacrylate monomers, styrene acrylics, carboxylate acrylics, and other similar units. Molecular weights reach > 200,000 g·mol^−1^, although some of them contain shorter chains (~8500 g·mol^−1^) [26,27,28,29]. Latex might also contain other minor components such as ammonia, polyethylene and silicon-based antifoam [27]. Interestingly, different acrylic additives have similar effects on the final properties of CNT films, according to the literature [26,27,28,29]. It can be deduced that the general action mechanism was nearly identical in all the cases. Moreover, other latex products, such as water-borne polyurethane and rubber resins, might also behave in analogous ways, given their equivalent compositions and chemical structures in terms of the transfer mechanism.

## 4. Conclusions

The addition of an acrylic component (Hycar^®^ 26084) to CNT conducting inks enables the fabrication of robust flexible electrodes on plastic substrates. Moreover, the electrodes are resistant to liquid contact, and thus are stable for electrochemical measurements. The concentration of the acrylic resin in the ink can be adjusted to selectively control the electrical and electrochemical properties of CNT film electrodes. Apart from tuning the electronic transport (electrical conductivity) and accumulation (capacitance), the acrylic component strongly blocks faradaic transfer reactions across the electrode–electrolyte interface. This blocking property might be relevant to avoid unspecific interactions, corrosion, and other parasitic reactions in electrodes or interfaces for analytical, sensing, and energy devices.

## Figures and Tables

**Table 1:** Labels and compositions (vol. %) of multi-walled carbon nanotube (MWCNT) liquid inks. Ratios refer to the three liquid components, namely, the MWCNT/sodium dodecylbenzenesulfonate (SDBS) dispersion (8 and 5 mg·mL^−1^, respectively, in water), commercial acrylic additive, and extra added water.

Label	MWCNT/SDBS	Hycar^®^	Water
MSH-0	100	0	0
MSH-1	50	1.6	48.4
MSH-2	50	3.1	46.9
MSH-3	50	6.3	43.8
MSH-4	50	12.5	37.5

**Table 2:** Labels and compositions (wt. %) of MWCNT inks on a dry basis.

Label	MWCNT	SDBS	Hycar^®^
MSH-0	61.5	38.5	0.0
MSH-1	30.0	18.7	51.3
MSH-2	19.8	12.4	67.8
MSH-3	11.8	7.4	80.8
MSH-4	6.5	4.1	89.4

**Table 3:** Thickness (*t*), mass (*m*), sheet resistivity (*R*_s_), electrical conductivity (*σ*), capacitance (*C*), and specific capacitance on a nanotube mass basis (*C*_s_) of MWCNT film electrodes.

Label	*t* (mm)	*m* (mg·cm^−2^)	*R*s (Ω·sq^−1^)	*σ* (S·cm^−1^)	*C* (mF·cm^−2^)	*C*_s_ (F·g^−1^)
MSH-0	0.01	0.874	12	83	8.0	14.88
MSH-1	0.07	6.492	4.7	30	8.6	4.41
MSH-2	0.06	5.959	11	16	0.41	0.35
MSH-3	0.07	7.817	48	3.0	-	-
MSH-4	0.18	18.41	120	0.47	-	-

**Table 4:** Peak separation (Δ*E_p_*) and transfer kinetic constant (*k*^0^) for ferricyanide and hydroquinone probes on MSH-0 and MSH-1 electrodes.

Label	Redox Probe	Δ*E_p_* (mV)	*k*^0^ (cm·s^−1^)
MSH-0	ferricyanide	151	1.84 × 10^−3^
MSH-0	hydroquinone	164	9.63 × 10^−3^
MSH-1	ferricyanide	350	1.62 × 10^−4^
MSH-1	hydroquinone	706	3.82 × 10^−6^
